# Supplementary material for: The Evolution and Characterization of the RNA Interference Pathways in Lophotrochozoa
Source: Genome Biol Evol. 2024 May 7;16(5):evae098. doi: 10.1093/gbe/evae098 (PMC11114477; doi:10.1093/gbe/evae098)
Supplement: evae098_Supplementary_Data [file evae098_supplementary_data.zip › Supplementary_materials.zip!/selected_miRNAs/NoGe_selected_miRNAs/known_miRNA_families_pdf_files/NMRB01000380.1_Notospermus_geniculatus_strain_Ushimado_scaffold380,_whole_genome_shotgun_sequence_11925.pdf]

Provisional ID : NMRB01000380.1_Notospermus_geniculatus_strain_Ushimado_scaffold380,_whole_genome_shotgun_sequence_11925
Score total : 2447.3
Score for star read(s) : 3.9
Score for read counts : 2438.6
Score for mfe : 1.8
Score for randfold : 0
Score for cons. seed : 3
Total read count : 4795
Mature read count : 4483
Loop read count : 0
Star read count : 312

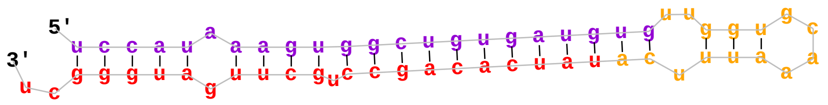

Genome coordinates: NMRB01000380.1 234632-234692

pre-miRNA sequence:
UCCAUAAAGUGGCUGUGAUGUGUCCAUAAAGUGGCUGUGAUGUGUUGGUGCAAAUUUCAUAUCACAGCCUGCUUGAUGGGCU

Mature sequence: UAUCACAGCCUGCUUGAUGGGCU

Aligned mature sequence of the *N. geniculatus* pre-miRNA with the top 5 hits from the BLAST search against the pre-miRNA mirGeneDB set:

```
                            ***************
Nge_Mir-2-o73               uaucacagccugcuugaugggc-u
Ovu-Mir-2-o21_3p            uaucacagccugcuuggaucagua
Obi-Mir-2-o24_3p            uaucacagccugcuuugaugag-a
Ovu-Mir-2-o24_3p            uaucacagccugcuuugaugag-a
Npo-Mir-2-o21_3p            uaucacagccugcuuggaucagua
Lan-Mir-2-o73_3p            uaucacagccugcuuugaugag-a
```
